# Supplementary material for: Sulfated polysaccharides from Phaeodactylum tricornutum: isolation, structural characteristics, and inhibiting HepG2 growth activity in vitro
Source: PeerJ. 2019 Feb 19;7:e6409. doi: 10.7717/peerj.6409 (PMC6385690; doi:10.7717/peerj.6409)

Apoptosis：

CK:


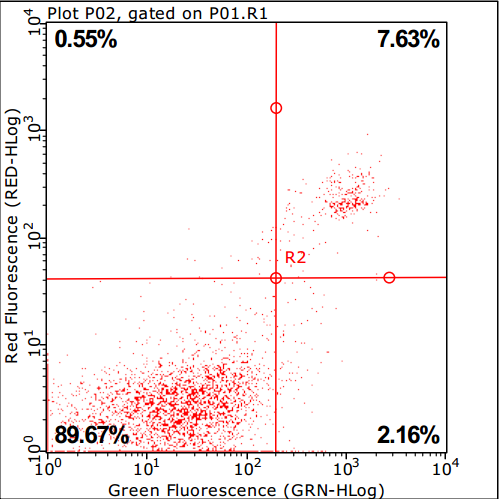


PTP 50ug/ml:


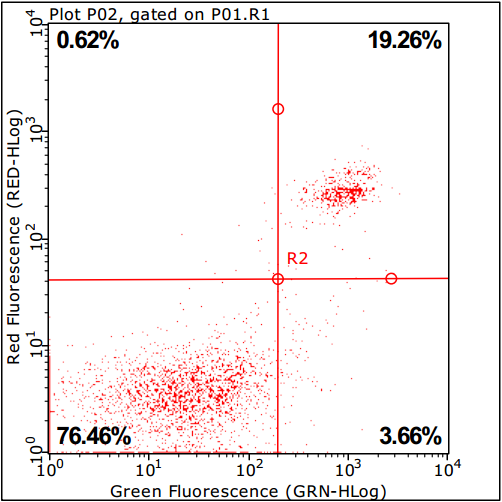


PTP 100ug/ml:


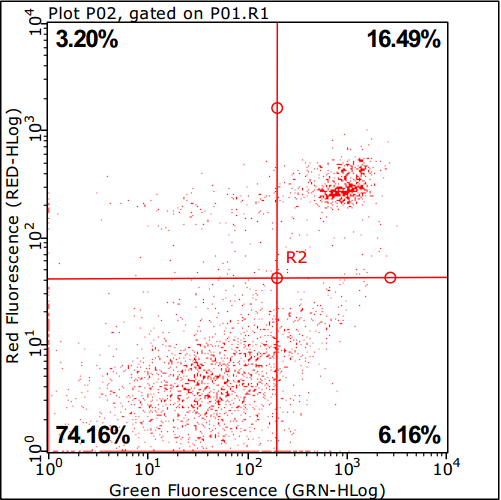


PTP 150ug/ml:


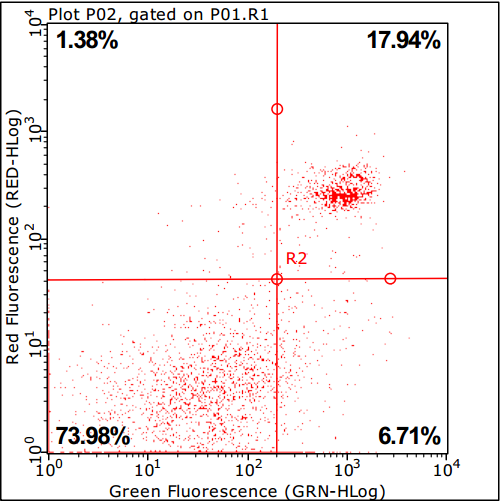


PTP 200ug/ml:


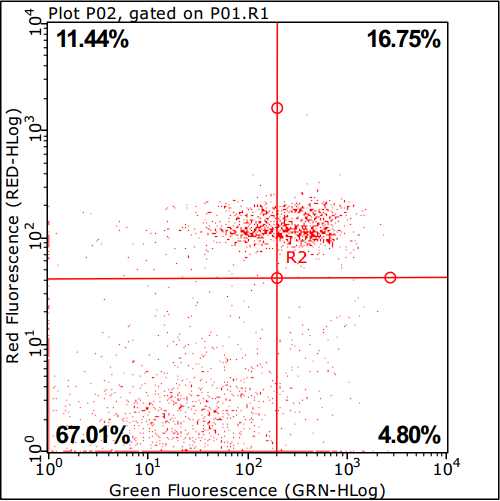


PTP 250ug/ml:


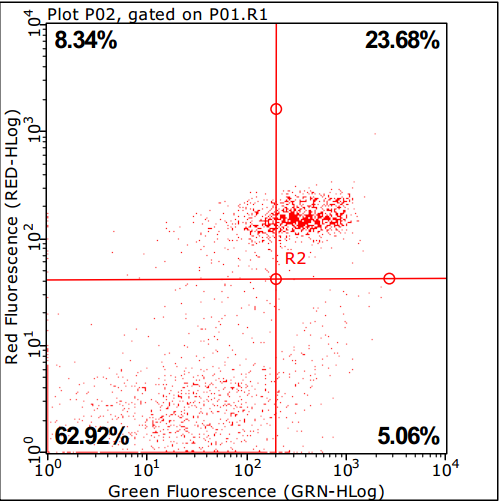


Cycle analysis:

CK:


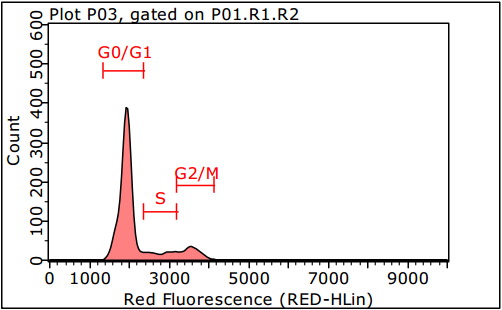


PTP 50ug/ml:


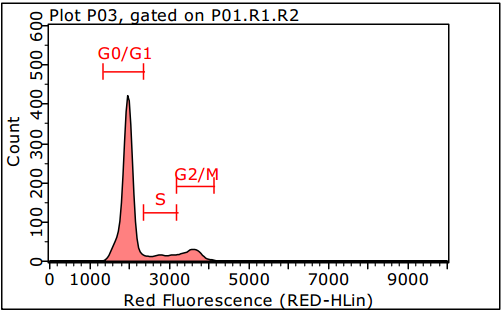


PTP 150ug/ml:


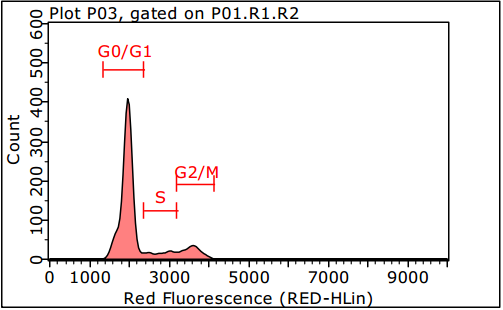


PTP 250ug/ml:


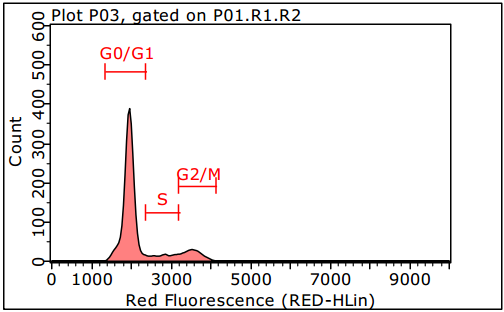

Supplement: Supplemental Information 2 [file peerj-07-6409-s002.doc]
