# Supplementary material for: Sulfated polysaccharides from Phaeodactylum tricornutum: isolation, structural characteristics, and inhibiting HepG2 growth activity in vitro
Source: PeerJ. 2019 Feb 19;7:e6409. doi: 10.7717/peerj.6409 (PMC6385690; doi:10.7717/peerj.6409)
Supplement: Supplemental Information 3 — Selecting the optimal extraction conditions [file peerj-07-6409-s003.docx]

**Extraction conditions:**

**Table 1 The optimum temperature**

| **Temperature(**℃) | **The yield of PTP** |
| --- | --- |
| 70 | 1.03% |
| 80 | 1.32% |
| 90 | 0.77% |

**Table 2 The optimum times of ultrasonic treatment(380w, 10s work, 10s rest)**

| **Times** | **The yield of PTP** |
| --- | --- |
| 5 | 0.42% |
| 10 | 0.86% |
| 20 | 1.32% |
| 40 | 0.77% |
| 60 | 1.29% |

**Table 3 The optimum extraction time**

| **Extraction time(h)** | **The yield of PTP** |
| --- | --- |
| 2 | 1.52% |
| 4 | 1.32% |
| 6 | 0.86% |
